# Feasibility Assessment of Using Geoinformatics Technology in Disaster Disease Surveillance in a Developing Country, Iran

**DOI:** 10.1371/currents.dis.cbb7c1d062d4ec0646c2d67319a312f2

**Published:** 2015-04-14

**Authors:** Md Omar Faruque, Kourosh Holakouie Naieni, Ali Ardalan, Elham Ahmadnezhad, Leila Mohammadinia

**Affiliations:** International Campus (Pardis), Tehran University of Medical Sciences, Tehran, Iran; Department of Epidemiology and Biostatistics, School of Public Health, Tehran University of Medical Sciences, Iranian Epidemiological Association, Tehran, Iran; Department of Disaster & Emergency Health , School of Public Health, Tehran University of Medical Sciences, Tehran, Iran; Department of Disaster & Emergency Health, National Institute of Health Research, Tehran University of Medical Sciences, Tehran, Iran; Harvard Humanitarian Initiative, Harvard University, Cambridge, USA; Department of Disaster & Emergency Health , School of Public Health, Tehran University of Medical Sciences, Tehran, Iran; Department of Disaster & Emergency Health, National Institute of Health Research, Tehran University of Medical Sciences, Tehran, Iran; Department of Disaster & Emergency Health , School of Public Health, Tehran University of Medical Sciences, Tehran, Iran

## Abstract

Background and purpose: Geoinformatics technology retains an unprecedented trait of performing with a supersonic speed and precision in public health management whereas the existing disease surveillance systems in developing countries lack using this technology. This article aims to assess the feasibility of using geoinformatics technology in disaster disease surveillance in a developing country, Iran. Methods: A self-administered questionnaire was developed based on technology acceptance model (TAM), and a semi-quantitative survey was conducted in order to collect data. Fifty TUMS & HS personnel, currently involve in disease surveillance and information technology, were included. Initially, a pilot study was conducted to test the validity and reliability of the questionnaire. Cronbach alpha, confirmatory factor analysis (CFA), and standard error of measurement (SEM) were calculated to validate the causal model. Results: The results from structural equation analysis suggested that TAM provided a constructive picture of using geoinformatics technology in disaster disease surveillance at TUMS &HS. The study found attitude (ATT) had a significant influence on participants intention to use (ITU) a new technology, and perceived ease of use (PEOU) was a strong determinant of perceived of usefulness (PU). Subsequently, PU and PEOU explained ATT substantially; even though the analysis showed insignificant statistical association among these constructs. The high R2 (Coefficient of determination) of the constructs described respondents positive instinct towards accepting a new technology. Conclusion: The study reveals that personnel intent to adopt geoinformatics technology in disaster disease surveillance; and at the same time, they possess a positive attitude towards the technology. This study also found PEOU has a strong influence on PU, so information sessions and training on geoinformatics technology need to focus primarily on the applications and impacts of technology on disaster disease surveillance.

## Abbreviations

WHO – World Health Organization; CDC – Center for Disease Controls; TAM –Technology Acceptance Model; PU –Perceived of Usefulness; PEOU –Perceived Ease of Use; ATT –Attitude; ITU –Intention to Use; TUMS –Tehran University of Medical Sciences; HS – health System; SEM –Standard Error of Measurement; ICC –Intra-class Correlation Coefficient; GFI –Goodness of Fit; CFI –Comparative Fit Index; NFI –Normed Fit Index; RMS –Root Mean Square

## Introduction

Surveillance concept emerged with the development of public health in the Western world in the 17th century. Initially, the surveillance system was confined counting death and diseases. In 19th century, efforts were directed to nationalize health information, collect vital statistics, perform analysis, and reporting mechanism. In twenty-first world health assembly--1968, the World Health Organization (WHO) integrated surveillance with disaster management along with other public health issues.^1^ Before that, a rapid surveillance was successfully applied to assess the nutritional status and identify population in need during Nigerian civil war in 1957.^2^

Most of the developed countries and advanced public health organizations incorporated disaster surveillance, with a view to reduce morbidity and mortality during and after calamities. For instance, The American Red Cross and Centers for Disease Controls (CDC) established a natural disaster surveillance system in 1987.^3^ Following hurricane Katrina in 2006, CDC developed Disaster Surveillance Work Group to co-ordinate surveillance activities, and evaluates existing tools and methods for assessing human health status.^4^^,^ 5 Recently, WHO approved a resolution on, “strengthening national health emergency and disaster management capacities and resilience of health systems” in order to strengthening the disaster surveillance system worldwide.^6^

Islamic Republic of Iran experiences frequent natural disaster due to its geographical location.^7^ Sistan and Baluchestan earthquake, 2013 (magnitude 7.7), Bam earthquake, 2003 (magnitude 6.6), Qayen earthquake, 1997 (magnitude 7.3), and cyclone Gonu (2007) are some devastating disasters in the history of the country.^8^^,^ 9 ^,^ 10 ^,^ 11 In last four decades, disasters caused 109,000 deaths, affected 53 million populations, and displaced tens of thousands of people. Iran scored 8 of 10 in disaster risk level and ranked 2nd in the world possessing 1,073,366 long standing refugees in 2011.^12^

Technology acceptance model (TAM) is a well-established, widely accepted, and frequently applied research model.^13^^,^ 14 TAM analyzes four human intuitions toward technology acceptance: perceived usefulness (PU), Perceived Ease of Use (PEOU), Attitude (ATT), and Intention to Use (ITU) towards a new technology.^13^ Davis (1989) defined PU as, “the degree to which a person believes that using a particular system would enhance his or her job performance,” and PEOU as, “the degree to which a person believes that using a particular system would be free of effort”.^15^ Davis et al (1989) defined attitude as, “the degree to which an individual evaluates and associates the target system with his or her job”.^16^ In this study, intention to use of geoinformatics technology operationalized as an individual extent of willingness to use geoinformatics technology in disaster disease surveillance.

The primary objective of this study is to foresee the feasibility of using geoinformatics technology that includes: Geographical Information System (GIS), Geographical Positioning System (GPS), and Remote Sensing in disaster disease surveillance in a developing country like Iran. The study conducted at TUMS & HS that provides health care services to 2.5 million population, examining personnel’ four perceptions: PU, PEOU, ATT, and ITU towards adopting geoinformatics technology with existing surveillance system. To accomplish this purpose, a cross sectional study was conducted to understand personnel intention towards acceptance of geoinformatics technology in disaster disease surveillance context; TAM has been used as analytic model.

## Materials and methods

**Study design**

A survey was conducted in 2014. By keeping other relevant variables constant, four constructs (PU, PEOU, ATT, ITU) containing 11 questions were used to assess the feasibility of integrating geoinformatics technology with existing disease surveillance system.

**Questionnaire development and pretest**

The main model and constructs were prepared according to the procedure outlined by Davis (1989).^15^ The items under each construct were adapted from existing literatures relevant to this study, 17 ^-^ 19 and a multi-item scale based on seven Point Likert-type scales (1. Strongly agree 2. Moderately agree 3. Agree 4. No opinion 5. Disagree 6. Moderately disagree 7. Strongly disagree) was added to suit the context of the study.^20^

After finalizing the model with its constructs and underlying items, the preliminary questionnaire was translated into Farsi by a native research expert. Then the questionnaire was reviewed by four experts in Farsi language. To examine the face validity, the questionnaire was sent to 10 disease surveillance experts: and they were requested to give their valuable opinion regarding simplicity, relevancy, and clarity about each question. The same experts were also entreated to provide their opinion regarding necessity of each question in order to evaluate questionnaire content validity. Each question was categorized into three levels- necessary, useful but not necessary, and not necessary. Finally, the questions were modified according to their suggestions.

Test-retest method was applied to evaluate the reliability of the questionnaire. Twelve PhD students from Disaster Management Academia, TUMS, participated in the pilot study. Two samples were taken in an interval of one week; Cronbach alpha, intra-class correlation coefficient (ICC), and standard error of measurement (SEM) were calculated. “If item deleted” option was applied to measure the Cronbach alpha and the “cut off” level was 0.70; Cronbach alpha values were well above the acceptable level.^21^ Items contained negative ICC value was deleted while remaining positive values varied from 0.20 to 0.74. SEM was calculated according to method described by Weir (2005).^22^ SEM ranged from 0.98 to 1.45 which revealed a small error of measurement.

The final questionnaire possessed two sections. Opening part consisted of participants’ demographic items such gender, educational level, field of specialization, training related to information technology, and job experience. The second segment contained questions that qualified in the pilot study.

**Sample size and sampling method**

Self-administered questionnaire and convenient sampling method were used to collect data. Participants included 50 personnel from three District Health Centers under TUMS&HS. Twenty six from South Tehran District Health Center, 14 from Islamshahr District Health Center, and 10 samples from Rey District Health Center were collected.

**Study area**

The survey was conducted in three District Health Center under Tehran University of Medical Sciences. South Tehran District Health Center (Jonoab), Eslamshahr District Health Center, and Rey District Health Center were the sites surveyed in January 2014. The criteria for selecting the subjects were: personnel currently engage in disease surveillance (communicable and non- communicable diseases), and personnel who look after disease surveillance related information technology from each of three districts. Staffs below this level were excluded.

**Statistical analysis**

Descriptive statistical analysis was performed to show demographic characteristics of the participants. Cronbach alpha, confirmatory factor analysis, and goodness of fit were calculated for measurement model, and multiple regression analysis was performed in structural equation model. SPSS._20_ (IBM Corp. Released 2011.IBM SPSS Statistics for Windows, Version 20.0. Armonk, NY: IBM Corp) was used for descriptive statistics and Cronbach alpha, while SPSS Amos._20_ (IBM Corp. Released 2011.IBM SPSS Statistics for Windows, Version 20.0. Armonk, NY: IBM Corp) was applied in performing confirmatory factor analysis, goodness of fit, and structural equation model.

## Results

Fifty samples were collected during survey. Although most of the questionnaires were filled thoroughly by the respondents, some questionnaires were with some missing values. Mean value was used to handle the missing values. Table-1 shows the demographic characteristics of the sample population.

**Table 1: Participants' demographics (N=50):** 

Characteristics	Number (%)
**Gender ** ****	
Male	18 (36%)
Female	32 (64%)
**Level of Education**	
Bachelor	46 (92%)
Master’s	3 (6%)
PhD	1 (2%)
**Field of Specialization**	
Health	43 (86%)
Information Technology	5 (10%)
Others	2 (2%)
**Training related to Information Technology**	
Yes	30 (60%)
No	20 (40%)
**Job related experience **	
1 to 5 years	16 (32%)
6 to 10 years	5 (10%)
11 to 15 years	9 (18%)
16 to 20 years	13 (26%)
21 to 25 years	4 (8%)
26 years and above	3 (6%)

About two third of the respondents took part in the survey are women. Three-fourth of the participants in the South Tehran and Eslamshahr centers are female, and an equal gender distribution found in the Rey Health District. A large majority of the respondents completed their bachelor degree while a smidgen participant holds master or PhD degree. Data reveals that same educational pattern exist in all three District Health Center.

A similar distribution prevails in the field of specialization where more than three quarter of the partaker expert in Public Health whereas less than 15% respondents have specialization in Information Technology and other related field. Three-fifth of the surveyed officers possesses training related to information technology. The DHC share the same IT training distribution except South Tehran district health center where the percentage is below than that of general finding. More than one third of the participants have longer job experience (16 to 20 years); same portion of the respondent is new starter (1 to 5 years). The most experience personnel occupy the least space in the field.


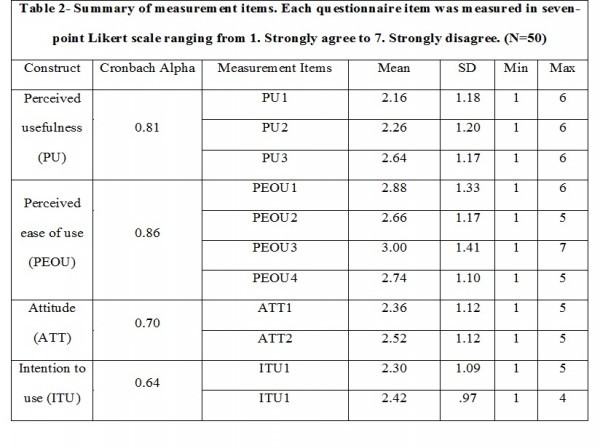


Table-2 shows the descriptive statistics of the four constructs along with Cronbach alpha value. Three of the constructs possess Cronbach alpha value well above 0.70 while one values 0.64 which is within acceptable level. Mean of each question falls below three (Likert scale) that indicates participants were consistent in their responses. Standard deviation ranges from 0.97 to 1.41 which reflects a narrow range of variability, and responses vary widely from one to six.

**Table 3: Goodness of fit measures:** 

Fit measures	Values
Chi squared	87.232
RMR (root mean square residual)	0.117
RMSEA (root mean square error of approximation)	0.115
GFI( goodness of fit index)	0.754
CFI (comparative fit index)	0.847
NFI (normed fit index)	0.760

The validated model contains 15 items including 11 questions and four constructs. The chi-square statistic is a strong indicator of goodness of fit for data and proposed model. Table-3 shows a relative good of fitness; chi-square, (N=50) = 87.232, degree of freedom= 40, p<0.001. Other goodness of fit statistics was also considered to examine fit indices of the model. Goodness of fit (GFI), comparative fit index (CFI), and normed fit index (NFI) are remarkable if exceed 0.90, but acceptable if close or above 0.80. Root mean square (RMS) acceptable value is within 0.5-0.8. The fit indices show that the study model does not contain the values within range due to small study sample but close enough to progress next stage examining path coefficients.

**Table 4: Confirmatory factor analysis. Factor loading:** 

Items	Perceived usefulness (PU)	Perceived ease of use (PEOU)	Attitude (ATT)	Intention to use (ITU)
PU1	0.75			
PU2	0.76			
PU3	0.79			
PEOU1		0.78		
PEOU2		0.77		
PEOU3		0.77		
PEOU4		0.82		
ATT1			0.77	
ATT2			0.71	
ITU1				0.64
ITU2				0.74

Table-4 represents confirmatory factor analysis. Confirmatory factor analysis also indicates the goodness of fit of the proposed model. Only one item takes the value below 0.70; otherwise, rest of the items partake value well above acceptable level.

**Table 5: Hypothesis Testing:** 

Hypothesis	Pathway	Path coefficient	t-value	p-value	Results
H1	PEOU → PU	0.897	5.268	*** ****	**Supported **
H2	PEOU → ATT	0.418	1.376	0.169	Not supported
H3	PU → ATT	0.408	1.346	0.178	Not supported
H4	ATT → ITU	0.803	3.751	***	**Supported **

Table-5 shows the final outcome of the TAM. Multiple regression analysis based on structural equation model was used to analyze the hypotheses.

**The validated Technology Acceptance. R2= Coefficient of determination.**
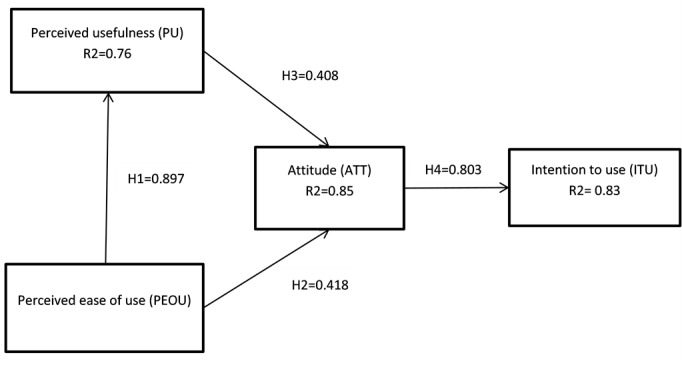

Figure 1 illustrates two hypotheses are significant. PEOU significantly correlated with PU, and ATT strongly associated with participants’ ITU whereas relationships between PEOU and ATT, and PU and ATT found statistically insignificant. The regression coefficient of H1 is 0.90, t-statistic 5.27, and p-value < 0.001 which confirms strong impact of PEOU on PU. H4 pathway contains regression weight 0.803, t- value 3.731, and p-score <0.001 that also supports a strong relationship between participants attitude towards intention to use. Other path (H2&H3) coefficients do not support the remaining hypotheses.

PEOU explains PU substantially. PEOU contributes about 90% variability of PU, while PEOU and PU together explain 85% variability of participants’ attitude. ATT also describes the ITU extensively (83%).

## Discussion

This study found significant statistical relationship between PEOU and PU, and between ATT and ITU. This study also revealed a high R^2 ^(coefficient of determination) of the constructs.

TAM had been used widely evaluating consumers perception in technology acceptance in recent years. Since the model predicts users’ perception towards using a technology with a high precision,^14^ so a number of authors used this model successfully. Health experts also used TAM to forecast users’ intention towards a new technology intervention in a number of studies, which shows the validity and usefulness of TAM model. Physician’s intention to use telemedicine in technology in Hong Kong; physiotherapists’ acceptance of a low cost portable system for postural assessment; patients’ acceptance of provider delivered e-health; public health nurses’ intention towards web based learning; mobile computing acceptance factors in healthcare and nurses’ intention of adopting an electronic logistic information system in Taiwan, are good examples of using TAM in assessing users acceptance towards new technology.^20^^,^ 23 ^-^ 26 So, applying TAM evaluating participants’ intention towards geoinformatics technology in disaster surveillance was acceptable.

This study found a significant association between PEOU and PU. According to Szajna and Bernadette (1996), when users hold good understandings about a particular technology, they become less reluctant to the usefulness of technology.^27^ The results indicate the participants were of the view that if geoinformatics technology is easy enough to use in disaster disease surveillance then it would be more useful in practice. Therefore, the response of the participants may be considered as valid document because it is supported by previous published studies.^18^^,^ 19 ^,^ 28 Sun and Zhang (2006) in their review found that 86% of the studies support a strong relationship between PEOU and PU.^29^ Furthermore, the study determined a significant statistical relationship between ATT and ITU. A high influence of ATT over ITU was observed as R^2 ^(coefficient of determination) value is 0.83, which means the study participants’ belief explains 83% variability of their intention to use geoinformatics. Positive attitude towards adopt a new technology is consistent with several other TAM studies.^18^^,^ 20 ^,^ 30 ^-^ 32

Even though this research found an important association between ATT and ITU, but the result is unprecedented with previous studies. Sun and Zhang (2006) have reviewed 21 studies carried on technology acceptance model and they found that more than 20% of the studies do not support a significant association between attitudes toward users’ intention to use of a technology.^29^ Mathieson (1991) has described that availability and market price of technology may have an influential role to raise consumers’ belief toward technology acceptance.^30^ Personal attitude is responsible for individual belief which in turn influences towards technology use.^16^ The study result reflects that personnel, currently engage in disease surveillance at TUMS, strongly believe that using geoinformatics technology in disaster disease surveillance is feasible in current settings.

This study found no significant relationship with perceived usefulness (PU) and attitude, and between perceived ease of use (PEOU) with attitude. Together, POEU and PU explained 85% variability of attitude which is more than that of reported by Taylor and Todd (1995), and Mathieson (1991) 73%.^30^^,^ 33 Empirically, perceived usefulness (PU) outclasses perceived ease of use (PEOU), because users investigate the usefulness and profit of a technology. If technology improves the daily activities of consumers, they prefer to utilize that technology instead of associated technical difficulties.^15^^,^ 18

Although the research showed harmony with the previous studies regarding association of PEOU and ATT, the study was inconsistent in relation to PU and ATT. Sun and Zhang (2006) have found in their review approximately 34% negative association between PEOU and ATT, while this relation was only 4% between PU and ATT.^29^ This may be the cause of lesser TAM applicability or fewer sampling or combination.

Personnel currently working in the disease surveillance ranging from novice to 25 years’ of experience, and three - fifth of the employees involved in surveillance for more than 10 years. They have become habituated working with a specific system for a long time, and suddenly adapting another technology could be challenging for them. Significant number of employees has no practical training in information technology, and it might also be the cause of their reluctance to adapt a new technology in their routine service. Low enthusiasm could be another possible reason not to learn the technology. Though, this study showed together PEOU and PU explained significant variability of attitude, but demonstrated no significant association with ATT individually.

## Limitations of the study

Even though the study reveals a positive intention towards adopting geoinformatics technology in disaster disease surveillance among TUMS & HS personnel, but the technology could be non-applicable both in other health set up within countries and in other developing countries. The personnel involved in disaster surveillance at different health care center in Iran may reluctant to integrate the technology in question with the existing system due to lack of technical skills and expertize over such high technology. In other developing countries may unable to adopt the technology in disaster surveillance owing to inadequate skilled personnel, lack of technology, insufficient funding, and even want of government interest.

Empirically, TAM model possess some drawbacks. This study based on individual response which means the participants provided their opinion according their own faith and belief, so responses could differ significantly among respondents. Also, the research result failed to show association between PEOU and ATT, and PU and ATT which indicates low validity and reliability of the tool used in data collection. As the instrument unable to measure relationship among constructs precisely, so it reflects the “non-applicability” of the instrument using in different context. Additionally, the study might fail to catch the overall complexity of using geoinformatics technology in disaster disease surveillance. Moreover, the study sample size was relatively small and it only focused on the specific subject group; the ratio of disease surveillance officer and personnel was disproportionate.

Longitudinal study, an objective procedure, could be a good option to minimize those study handicaps. Extended technology acceptance model could also be a more reliable method in this study, since extended TAM considers other factors like age, work experience, race, education, and other relevant variables to explain the feasibility of a technology acceptance. Inclusion of gender, educational level, field of specialization, IT training, job experience into the model would give more valid and reliable results. This study could be considered as an initiation of assessing feasibility of using geoinformatics technology with existing disease surveillance system.

## Conclusion

This study set out to foresee users acceptance of geoinformatics technology in disaster disease surveillance in a developing country, and to analyze the interrelationships among four variables; PU, PEOU, ATT, and ITU. It was expected to have close relationships among the variables; that’s why, TAM was applied. The research also directed to test how significant the TAM in explaining user satisfaction and intention to use geoinformatics technology. For instance, the study found a strong statistical association between ATT and ITU.

From a practical standpoint, this study is meaningful because findings reveal that personnel intent to adopt geoinformatics technology in disaster disease surveillance; and at the same time, they also possess a positive attitude towards the technology. Positive attitude is required in order to foster individual intentions to use a technology; it is also important to encourage and cultivate a positive attitude toward using the technology. Additionally, geoinformatics technology should improve the effectiveness and efficacy of the disaster disease surveillance because users prefer technology’s usefulness before ease to use. Moreover, the study result found PEOU has a strong influence on PU, so information sessions and training on geoinformatics technology need to focus primarily on how the technology can help to improve the efficiency and effectiveness of disaster disease surveillance rather than on the steps or procedures of actual use of the technology .

Future research efforts are needed to address the limitations of this study. Initially, effort needs to direct to expand the theoretical validity of the literature, re-examination of ΤΑΜ with different IT set up will be important. Ideally, this should be a series of studies in a variety of contexts including different technology and professional users over a period of time. Finally, this study did not incorporate demographic variables like age, IT training, or work experience into the model. So, an extended TAM which integrates demographic variables in the analysis could be used to access more reliable and valid results.

## Competing Interests

The authors have declared that no competing interests exist.

## Appendix 1

### Measurement Items Used in the Study

Perceived Usefulness (PU)

PU1: Using geoinformatics technology can increase productivity of disaster disease surveillance.

PU2: Using geoinformatics technology will save organizational time and money in disaster disease surveillance.

PU3: Using geoinformatics technology will allow more efficient distribution of resources during disaster management.

Perceived ease of use (PEOU)

PEOU1: learning to operate geoinformatics system in disaster disease surveillance will be easy for me.

PEOU2: I would find it easy get geoinformatics technology in disaster disease surveillance system to do what I want it to do.

PEOU3: My interaction with using geoinformatics technology in disaster disease surveillance system would be clear and understandable.

PEOU4: I would find using geoinformatics technology in disaster disease surveillance system to be flexible to interact with.

Attitude (ATT)

ATT1: I am completely satisfied using geoinformatics system in disaster disease surveillance.

ATT2: I am very confident in using geoinformatics system in disaster disease surveillance.

Intention to use (ITU)

ITU1: I believe that using geoinformatics system in disaster disease surveillance will improve the quality of the service.

ITU2: To the extent possible, I would use geoinformatics technology in disaster disease surveillance frequently.
